# Self-consistent determination of long-range electrostatics in neural network potentials

**DOI:** 10.1038/s41467-022-29243-2

**Published:** 2022-03-23

**Authors:** Ang Gao, Richard C. Remsing

**Affiliations:** 1 Department of Physics, Beijing University of Posts and Telecommunications, 100876 Beijing, China; 2 Department of Chemistry and Chemical Biology, Rutgers University, Piscataway, NJ 08854 USA

**Keywords:** Computational chemistry, Method development, Chemical physics, Molecular dynamics

## Abstract

Machine learning has the potential to revolutionize the field of molecular simulation through the development of efficient and accurate models of interatomic interactions. Neural networks can model interactions with the accuracy of quantum mechanics-based calculations, but with a fraction of the cost, enabling simulations of large systems over long timescales. However, implicit in the construction of neural network potentials is an assumption of locality, wherein atomic arrangements on the nanometer-scale are used to learn interatomic interactions. Because of this assumption, the resulting neural network models cannot describe long-range interactions that play critical roles in dielectric screening and chemical reactivity. Here, we address this issue by introducing the self-consistent field neural network — a general approach for learning the long-range response of molecular systems in neural network potentials that relies on a physically meaningful separation of the interatomic interactions — and demonstrate its utility by modeling liquid water with and without applied fields.

## Abstract (web summary)

Machine learning-based neural network potentials often cannot describe long-range interactions. Here the authors present an approach for building neural network potentials that can describe the electronic and nuclear response of molecular systems to long-range electrostatics.

## Introduction

Computer simulations have transformed our understanding of molecular systems by providing atomic-level insights into phenomena of widespread importance. The earliest models used efficient empirical descriptions of interatomic interactions, and similar force field-based simulations form the foundation of molecular simulations today^1^. However, it is difficult to describe processes like chemical reactions that involve bond breakage and formation, as well as electronic polarization effects within empirical force fields. The development of quantum mechanics-based ab initio simulations enabled the description of these complex processes, leading to profound insights across scientific disciplines^2–9^. The vast majority of these first principles approaches rely on density functional theory (DFT), and the development of increasingly accurate density functionals has greatly improved the reliability of ab initio predictions^10–15^. But, performing electronic structure calculations are expensive, and first-principles simulations are limited to small system sizes and short time scales.

The prohibitive expense of ab initio simulations can be overcome through machine learning. Armed with a set of ab initio data, machine learning can be used to train neural network (NN) potentials that describe interatomic interactions at the same level of accuracy as the ab initio methods, but with a fraction of the cost. Consequently, NN potentials enable ab initio quality simulations to reach the large system sizes and long time scales needed to model complex phenomena, such as phase diagrams^16–20^ and nucleation^21,22^.

Despite the significant advances made in this area, there are still practical and conceptual difficulties with NN potential development, especially with regard to long-range electrostatics. To make NN potential construction computationally feasible, most approaches learn only local arrangements of atoms around a central particle, where the meaning of “local” is defined by a distance cutoff usually <1 nm. Because of this locality, the resulting NN potentials are inherently short-ranged. The lack of long-range interactions in NN potentials can lead to both quantitative and qualitative errors, especially when describing polar and charged species^23–25^.

The need for incorporating long-range electrostatics into NN potentials has led to the development of several new approaches^23,24,26–29^. Many of these approaches exclude all or some of the electrostatic interactions from training and then assign effective partial charges to each atomic nucleus that are used to calculate long-range electrostatic interactions using traditional methods^23,25–28^. The values of these effective charges can be determined using machine learning methods. For example, the fourth-generation high-dimensional neural network potential (4G-HDNNP)^28^ employs deep NNs to predict the electronegativities of each nucleus, which are subsequently used within a charge equilibration process to determine the effective charges. These approaches can predict binding energies and charge transfer between molecules, but they also introduce quantities that are not direct physical observables, such as the effective charges and electronegativities. Another approach explicitly incorporated nonlocal geometric information into the construction of local feature functions^24,30^. This approach, referred to as the long-distance equivariant representation, is able to more accurately predict the binding energy between molecules and the polarizability of molecules, compared to purely local models. However, this model only takes in the coordinates of the nucleus as input information and cannot handle external fields.

The difficulties that current approaches to NN potentials have when treating long-range interactions can be resolved by a purely ab initio strategy that uses no effective quantities. Such a strategy can be informed by our understanding of the roles of short- and long-range interactions in condensed phases^31–34^. In uniform liquids, appropriately chosen uniformly slowly varying components of the long-range forces—van der Waals attractions and long-range Coulomb interactions—cancel to a good approximation in every relevant configuration. As a result, the local structure is determined almost entirely by short-range interactions. In water, these short-range interactions correspond to hydrogen bonding and packing^35–38^. Therefore, short-range models, including current NN potentials, can describe the structure of uniform systems. This idea, that short-range forces determine the structure of uniform systems, forms the foundation for the modern theory of bulk liquids^31–33^, in which the averaged effects of long-range interactions can be treated as a small correction to the purely short-range system.

In contrast, the effects of long-range interactions are more subtle and play a role in collective effects that are important for dielectric screening. Moreover, long-range forces do not cancel at extended interfaces and instead play a key role in interfacial physics. As a result, short-range models cannot describe interfacial structure and thermodynamics, as they do in the bulk, and standard NN models fail to describe even the simplest liquid-vapor interfaces^25^. The local molecular field (LMF) theory of Weeks and coworkers provides a framework for capturing the average effects of long-range interactions at interfaces through an effective external field^34,39–42^. LMF theory also provides physically intuitive insights into the roles of short- and long-range forces at interfaces that can be leveraged to model nonuniform systems.

Here, we exploit the physical picture provided by liquid-state theory to develop a general approach for learning long-range interactions in NN potentials from ab initio calculations. We separate the atomic interactions into appropriate short-range and long-range components and construct a separate network to handle each part. Importantly, the short-range model is isolated from the long-range interactions. This separation also isolates the long-range response of the system, enabling it to be learned. Short-range interactions can be learned using established approaches. The short- and long-range components of the potential are then connected through a rapidly converging self-consistent loop. The resulting self-consistent field neural network (SCFNN) model is able to describe the effects of long-range interactions without the use of effective charges or similar artificial quantities. We illustrate this point through the development of a SCFNN model of liquid water. In addition to capturing the local structure of liquid water, as evidenced by the radial distribution function, the SCFNN model accurately describes long-range structural correlations connected to dielectric screening, the response of liquid water to electrostatic fields, and water’s dielectric constant. Because the SCFNN model learns the response to electrostatic fields, it can predict properties that depend on screening in environments for which it was not trained. We demonstrate this by using the SCFNN trained on bulk configurations to model the orientational ordering of water at the interface with its vapor. Finally, the SCFNN also captures the electronic fluctuations of water and can accurately predict its high-frequency dielectric constant.

## Results

### Workflow of the SCFNN model

**Fig. 1:**
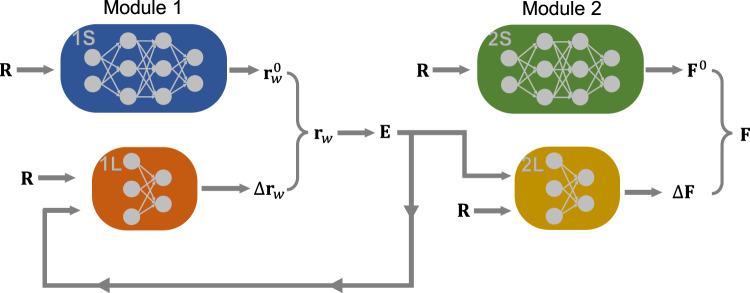
Schematic of the self-consistent field neural network (SCFNN). The SCFNN consists of two modules, each with two networks. One network learns the short-range interactions (S) and the other learns the effects of long-range interactions (L). Module 1 learns the positions of maximally localized Wannier function centers, **r**_*w*_, and Module 2 learns the forces, **F**, on the atomic nuclei, the positions of which are indicated by **R**.

The SCFNN model consists of two modules that each target a specific response of the system (Fig. 1). Module 1 predicts the electronic response via the position of the maximally localized Wannier function centers (MLWFCs). Module 2 predicts the forces on the nuclear sites. In turn, each module consists of two networks: one to describe the short-range interactions and one to describe perturbations to the short-range system from long-range electric fields. Together, these two modules (four networks) enable the model to predict the total electrostatic properties of the system.

In the short-range system, the *v*(*r*) = 1/*r* portion of the Coulomb potential is replaced by the short-range potential ${v}_{0}(r)={{{{{{{\rm{erfc}}}}}}}}(r/\sigma )/r$. Physically, *v*_0_(*r*) corresponds to screening the charge distributions in the system through the addition of neutralizing Gaussian charge distributions of opposite sign—the interactions are truncated by Gaussians. Therefore, we refer to this system as the Gaussian-truncated (GT) system^34–38^. By making a physically meaningful choice for *σ*, the GT system can describe the structure of bulk liquids with high accuracy but with a fraction of the computational cost. Moreover, the GT system has served as a useful short-range component system when modeling the effects of long-range fields^37,39,41,43,44^. Here, we choose *σ* to be 4.2 Å (8 Bohr), which is large enough for the GT system to accurately describe hydrogen bonding and the local structure of liquid water^34–38^.

The remaining part of the Coulomb interaction, ${v}_{1}(r)=v(r)-{v}_{0}(r)={{{{{{{\rm{erf}}}}}}}}(r/\sigma )/r$, is long ranged, but varies slowly over the scale of *σ*. Because *v*_1_(*r*) is uniformly slowly-varying, the effective field produced by *v*_1_(*r*) usually induces a linear response in the GT system. The linear nature of the response makes the effects of *v*_1_(*r*) able to be captured by linear models. In the context of NNs, we demonstrate below that a linear network is sufficient to learn the linear response induced by long-range interactions.

### Module 1

**Fig. 2:**
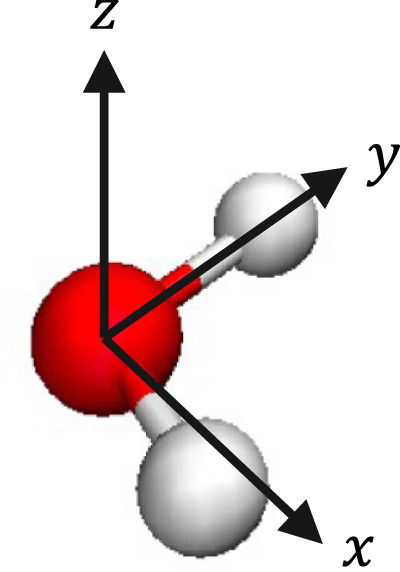
Local frame around a central water. The *y*-axis is along the OH bond. The *z*-axis is perpendicular to the plane of the molecule. The *x*-axis is perpendicular to the *x* and *z* axes.

The separation of interactions into short- and long-range components is crucial to the SCFNN model. In particular, the two networks of each module are used to handle this separation. Network 1S of Module 1 predicts the positions of the MLWFCs in the short-range GT system, while Network 1L predicts the perturbations to the MLWFC positions induced by the effective long-range field. Networks 1S and 1L leverage Kohn’s theory on the nearsightedness of electronic matter (NEM)^45,46^. The NEM states that^46^ “local electronic properties, such as the density *n*(*r*), depend significantly on the effective external potential only at nearby points.” Here the effective external potential includes the external potential and the self-consistently determined long-range electric fields. Therefore, the NEM suggests that the electronic density, and consequently the positions of the MLWFCs, are “nearsighted” with respect to the effective potential, but not to the atomic coordinates, contrary to what has been assumed in previous work that also uses local geometric information of atoms as input to NNs^47,48^. An atom located at ${{{{{{{\bf{r}}}}}}}}^{\prime}$ will affect the effective potential at **r**, even if ${{{{{{{\bf{r}}}}}}}}^{\prime}$ is far from **r**, through long-range electrostatic interactions. Consequently, current approaches to generating NN models can only predict the position of MLWFCs for a purely short-range system without long-range electrostatics, such as the GT system^47,48^. We exploit this fact and use established NNs to predict the locations of the MLWFCs in the GT system^47^. To do so, we create a local reference frame around each water molecule (Fig. 2) and use the coordinates of the surrounding atoms as inputs to the NN. The local reference system preserves the rotational and translational symmetry of the system. The network outputs the positions of the four MLWFCs around the central water, which are then transformed to the laboratory frame of reference.

Network 1L predicts the response of the MLWFC positions to the effective field **E**(**r**), defined as the sum of the external field, **E**_ext_(**r**), and the long-range field from *v*_1_(*r*):

1 $${{{\bf{E}}}} ({{{\bf{r}}}}) = {{{\bf{E}}}}_{{{\rm{ext}}}} ({{{\bf{r}}}}) - \int d{{{\bf{r}}}}^{\prime} \rho ({{{\bf{r}}}}^{\prime} )\nabla {v}_{1}(| {{{\bf{r}}}}-{{{\bf{r}}}}^{\prime}|)\,,$$

where $\rho ({{{{{{{\bf{r}}}}}}}}^{\prime} )$ is the instantaneous charge density of the system, including nuclear and electronic charges. Network 1L also introduces a local reference frame for each water molecule. However, Network 1L takes as input both the local coordinates and local effective electric fields. The NEM suggests that this local information is sufficient to determine the perturbation in the MLWFC positions. Network 1L outputs this change in the positions of the water molecule’s four MLWFCs, and this perturbation is added to the MLWFC position determined in the GT system to obtain the MLWFCs in the full system. We note that **E**(**r**) is a slowly-varying long-range field, such that the MLWFCs respond linearly to this field. Therefore, Network 1L is constructed to be linear in **E**(**r**). Table 1 demonstrates that the linear response embodied by Network 1L predicts the perturbation of the MLWFCs with reasonable accuracy.

**Table 1:** Mean Absolute Error (MAE), multiplied by 100, of Network 1L and 2L in predicting the changes in the maximally localized Wannier function center (MLWFC) positions (Å) and the forces (eV/Å) on the oxygen and hydrogen nuclei, *F*_O_ and *F*_H_, respectively, along the *z*-direction when fields of strength 0.1 and 0.2 V/Å are applied along the same direction.

	MLWFC positions	*F*_O_	*F*_H_
0.1 V/Å	0.028	1.4	0.98
0.2 V/Å	0.056	2.8	2.0

The predictions are made for the test sets and the error is computed with respect to the DFT results.

We now need to determine the effective field **E**(**r**). This effective field depends on the electron density distribution, but evaluating and including the full three-dimensional electron density for every configuration in a training set requires a prohibitively large amount of storage space. Instead, we approximate the electron density by the charge density of the MLWFCs, assuming each MLWFC is a point charge of magnitude −2*e*_0_. This approximation is often used when computing molecular multipoles, as needed to predict vibrational spectra, for example^14,48^. Here it is important to note that the MLWFs of water are highly localized so that the center gives a reasonable representation of the location of the MLWF. Moreover, the electron density is essentially smeared over the scale of *σ* through a convolution with *v*_1_(*r*), which makes the resulting fields relatively insensitive to small-wavelength variations in the charge density. As a result, the electron density can be accurately approximated by the MLWFC charge density within our approach.

The effective field is a functional of the set of MLWFC positions, ${{{{{{{\bf{E}}}}}}}}[\left\{{{{{{{{{\bf{r}}}}}}}}}_{w}\right\}]$, and the positions of the MLWFCs themselves depend on the field, **r**_*w*_[**E**]. Therefore, we determine **E** and $\left\{{{{{{{{{\bf{r}}}}}}}}}_{w}\right\}$ through self-consistent iteration. Our initial guess for **E** is obtained from the positions of the MLWFCs in the GT system. We then iterate this self-consistent loop until the MLWFC positions no longer change, within a tolerance of 2.6 × 10^−4^ Å. In practice, we find that self-consistency is achieved quickly.

### Module 2

After Module 1 predicts the positions of the MLWFCs, Module 2 predicts the forces on the atomic sites. As with the first module, Module 2 consists of two networks: one that predicts the forces of the GT system and another that predicts the forces produced by **E**(**r**). To predict the forces in the GT system, we adopt the network used by Behler and coworkers^49^. This network, Network 2S, takes local geometric information of the atoms as inputs and, consequently, cannot capture long-range interactions. To describe long-range interactions, we introduce a second network (Network 2L in Fig. 1). This additional network predicts the forces on atomic sites due to the effective field **E**(**r**), which properly accounts for long-range interactions in the system. In practice, we again introduce a local reference frame for each water molecule and use local atomic coordinates and local electric fields as inputs. In this case, we also find that a network that is linear in **E**(**r**) accurately predicts the resulting long-range forces, consistent with the linear response of the system to a slowly-varying field.

In practice, separating the data obtained from standard DFT calculations into the GT system and the long-range effective field is not straightforward. To solve this problem, we apply homogeneous electric fields of varying strength while keeping the atomic coordinates fixed. The fields only perturb the positions of the MLWFCs and the forces on the atoms—these perturbations are not related to the GT system. The changes induced by these electric fields are directly obtained from DFT calculations and are used to train Networks 1L and 2L and learn the response to long-range effective fields. The remaining part of the DFT data, which has the long-range field **E**(**r**) removed, is used to train Networks 1S and 2S and learn the response of the short-ranged GT system. See the “Methods” section for a more detailed discussion of the networks and the training procedure.

We emphasize that our approach to partitioning the system into a short-range GT piece and a long-range perturbation piece is different from other machine learning approaches for handling long-range electrostatics. The standard approaches usually partition the total energy into two parts, a short-ranged energy and an Ewald energy that is used to evaluate the long-range interactions. However, this partitioning results in a coupling between the short- and long-range interactions. For example, the short-range part of the energy in the 4G-HDNNP model depends on the effective charges that are assigned to the atoms, but these effective charges depend on long-range electrostatic interactions through the global charge equilibration process used to determine their values^28^. In contrast, the SCFNN approach isolates the short-range interactions during the training process and connects the short-range model to long-range interactions through **E**(**r**) via self-consistency. The GT system embodied by Network 1S and 2S does not depend on long-range electrostatics even implicitly; it is completely uncoupled from the long-range interactions. The effects of long-range electrostatic interactions are isolated within the second network of each module, Network 1L and Network 2L in Fig. 1. This separation of short- and long-ranged effects is similar in spirit to the principles underlying LMF theory^34,39,41^ and related theories of uniform liquids^32,33,35,36,38^.

### Water’s local structure is insensitive to long-range interactions

We demonstrate the success of the SCFNN approach by modeling liquid water. Water is the most important liquid on Earth. Yet, the importance of both short- and long-range interactions makes it difficult to model. Short-range interactions are responsible for water’s hydrogen bond network that is essential to its structure and unusual but important thermodynamic properties^36,50^. Long-range interactions play key roles in water’s dielectric response, interfacial structure, and can even influence water-mediated interactions^41,51^. Because of this broad importance, liquid water has served as a prototypical test system for many machine learning-based models^17,24,48,49,52^ Here, we test our SCFNN model on a system of bulk liquid water by performing molecular dynamics (MD) simulations of 1000 molecules in the canonical ensemble under periodic boundary conditions.

**Fig. 3:**
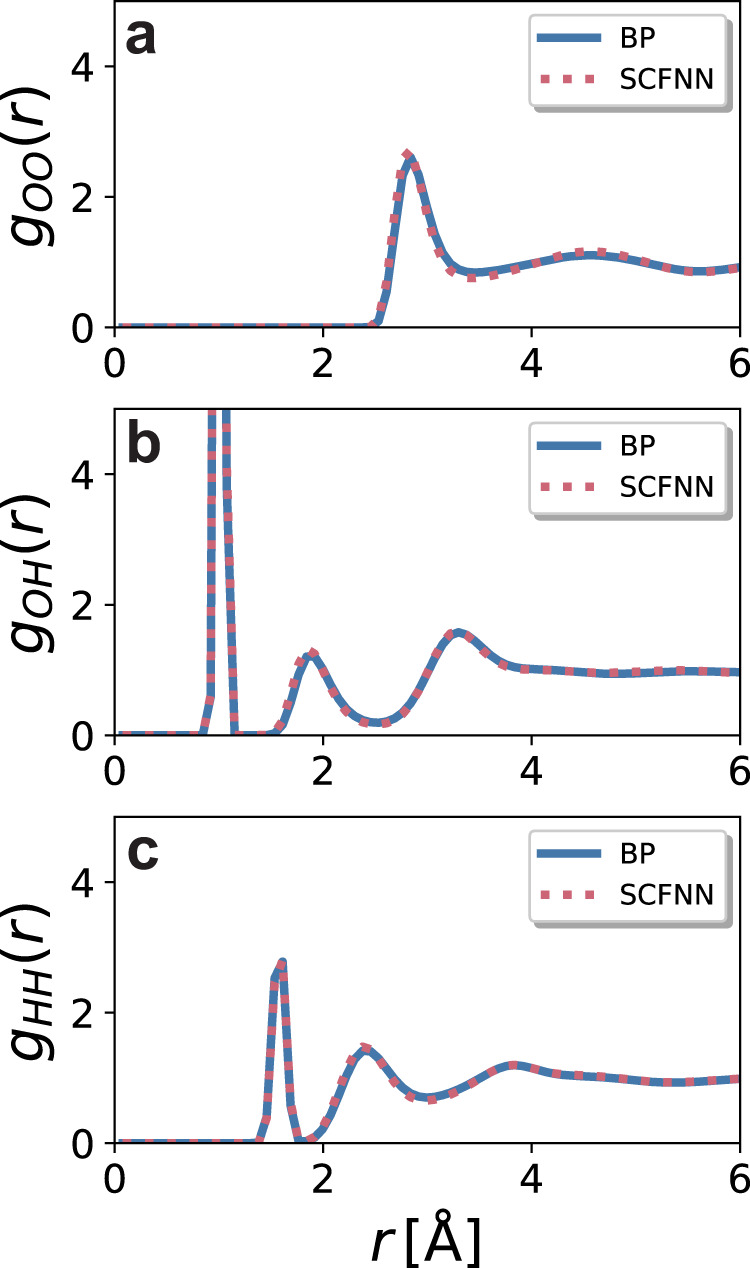
Local structure of bulk water. Comparison of the radial distribution functions for **a** O-O, **b** O-H, and **c** H-H correlations in liquid water, as predicted by molecular dynamics simulations of the self-consistent field neural network (SCFNN) and Behler–Parrinello (BP) models.

One conventional test on the validity of a NN potential is to compare the radial distribution function, *g*(*r*), between atomic sites for the different models. The *g*(*r*) predicted by the SCFNN model is the same as that predicted by the Behler–Parrinello (BP) model^49^ for all three site–site correlations in water (Fig. 3). This level of agreement may be expected, based on previous work examining the structure of bulk water^36–38,40,41^. The radial distribution functions of water are determined mainly by short-range, nearest-neighbor interactions, which arise from packing and hydrogen bonding; long-range interactions have little effect on the main features of *g*(*r*). Consequently, purely short-range models, like the GT system, can quantitatively reproduce the *g*(*r*) of water^36–38,40,41^. Similarly, the short-range BP model accurately describes the radial distribution functions, as does the SCFNN model, which includes long-range interactions.

### Long-range electrostatics and dielectric response

Though the short-range structure exemplified by the radial distribution function is insensitive to long-range interactions, long-range correlations are not. For example, the longitudinal component of the dipole density or polarization correlation function evaluated in reciprocal space, ${\chi }_{zz}^{0}({{{{{{{\bf{k}}}}}}}})$, was recently shown to be sensitive to long-range interactions^44^. This correlation function is defined according to

2 $${\chi }_{zz}^{0}({{{{{{{\bf{k}}}}}}}})=\frac{1}{V}\left\langle\mathop{\sum}\limits_{l,j}\frac{({{{{{{{\bf{k}}}}}}}}\cdot {{{{{{{{\bf{p}}}}}}}}}_{l})\,({{{{{{{\bf{k}}}}}}}}\cdot {{{{{{{{\bf{p}}}}}}}}}_{j})}{{k}^{2}}\,{e}^{-i{{{{{{{\bf{k}}}}}}}}\cdot \left({{{{{{{{\bf{r}}}}}}}}}_{l}-{{{{{{{{\bf{r}}}}}}}}}_{j}\right)}\right\rangle \,,\,\,{{{{{{{\rm{with}}}}}}}}\,\,{{{{{{{\bf{k}}}}}}}}=k\hat{{{{{{{{\bf{z}}}}}}}}}\,.$$

Here **p**_*j*_ is the dipole moment of water molecule *j* and **r**_*j*_ is the position of the oxygen atom of water molecule *j*.

Here we compare the longitudinal polarization correlation function predicted by our SCFNN model and the BP model. The original BP model is not able to predict molecular charge distributions. Therefore, to predict the dipole moment of water, we couple the BP model with the short-range part of the SCFNN model that predicts MLWFCs (Network 1S). We note that a similar strategy was used in the previous work^47^.

**Fig. 4:**
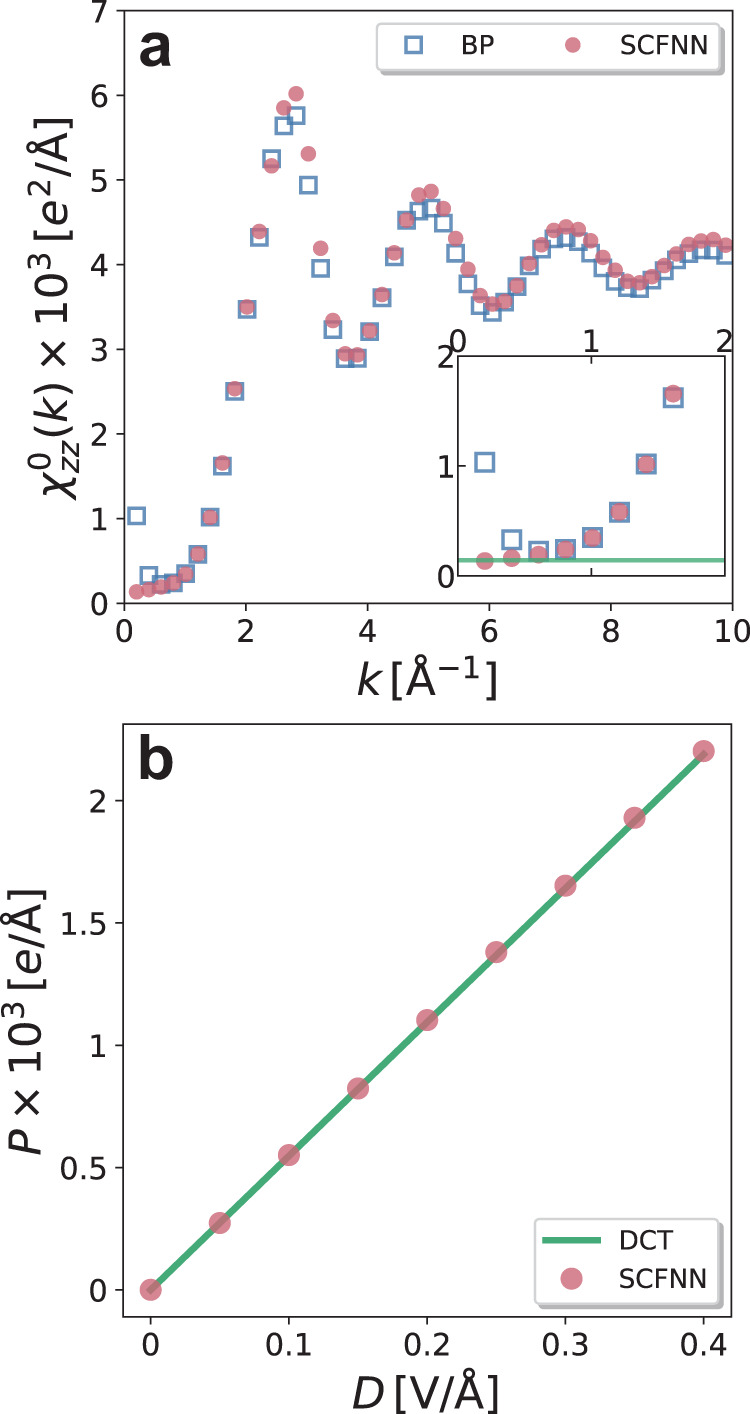
Long-range polarization in bulk water. **a** The longitudinal polarization correlation function in reciprocal space, ${\chi }_{zz}^{0}({{{{{{{\bf{k}}}}}}}})$, shows differences between the self-consistent field neural network (SCFNN) and Behler–Parrinello (BP) models at low *k*. In particular, the SCFNN model plateaus as *k* → 0 in a manner consistent with the theoretical prediction (green line), while the BP (short-range) model does not. **b** The polarization, *P*, induced by a homogeneous displacement field of magnitude *D* along the *z*-axis is accurately predicted by the SCFNN model, evidenced by the agreement with dielectric continuum theory (DCT) predictions.

The longitudinal polarization correlation function predicted by our SCFNN model and the BP agree everywhere except at small *k*, indicating that long-range correlations are different in the two models (Fig. 4a). The long-wavelength behavior of the polarization correlation function is related to the dielectric constant via^35,53,54^

3 $$\mathop{\lim }\limits_{k\to 0}{\chi }_{zz}^{0}({{{\bf{k}}}})={\varepsilon }_{0}{k}_{{{\rm{B}}}}T\frac{\varepsilon -1}{\varepsilon }\,,$$

where *ε* ≈ 100 is the value of the dielectric constant of water predicted by the SCFNN, as discussed below. The ${\chi }_{zz}^{0}({{{{{{{\bf{k}}}}}}}})$ predicted by our SCFNN model is consistent with the expected behavior at small *k*. In contrast, short-range models, like the GT system^35,44,54^ and the BP model, significantly deviate from the expected asymptotic value. Consequently, these short-range models are expected to have difficulties describing the dielectric screening that is important in nonuniform systems^25,37,39,41,44^, for example.

To further examine the dielectric properties of the NN models, we can apply homogeneous fields of varying strength to the system and examine its response. To do so, we performed finite-field simulations at constant displacement field, **D**. These finite-**D** simulations^55^ can be naturally combined with our SCFNN model, unlike many other NN models that cannot handle external fields. Following previous work^44^, we use ${{{{{{{\bf{D}}}}}}}}=D\hat{{{{{{{{\bf{z}}}}}}}}}$, vary the magnitude of the displacement field from *D* = 0 V/Å to *D* = 0.4 V/Å, and examine the polarization, *P*, induced in water. As shown in Fig. 4b, the polarization response of water to the external field is accurately predicted by dielectric continuum theory, as expected, further suggesting that the SCFNN model properly describes the dielectric response of water. To the best of our knowledge, this is the first NN model that can accurately describe the response of a system to external fields. We emphasize that this response is achieved by learning the long-range response via Networks 1L and 2L.

Because the SCFNN can predict the response to electrostatic fields, we can use a highly efficient method to estimate the dielectric constant^56^. To do so, we compute the *r*-dependent Kirkwood *g*-factor, *G*_K_(*r*), with **E** = 0 and **D** = 0, where

4 $${G}_{{{{{{{{\rm{K}}}}}}}}}(r)= \langle {{{{{{{{\boldsymbol{\mu }}}}}}}}}_{1}\cdot {{{{{{{{\bf{M}}}}}}}}}_{1}(r) \rangle /{\mu }^{2},$$

**Fig. 5:**
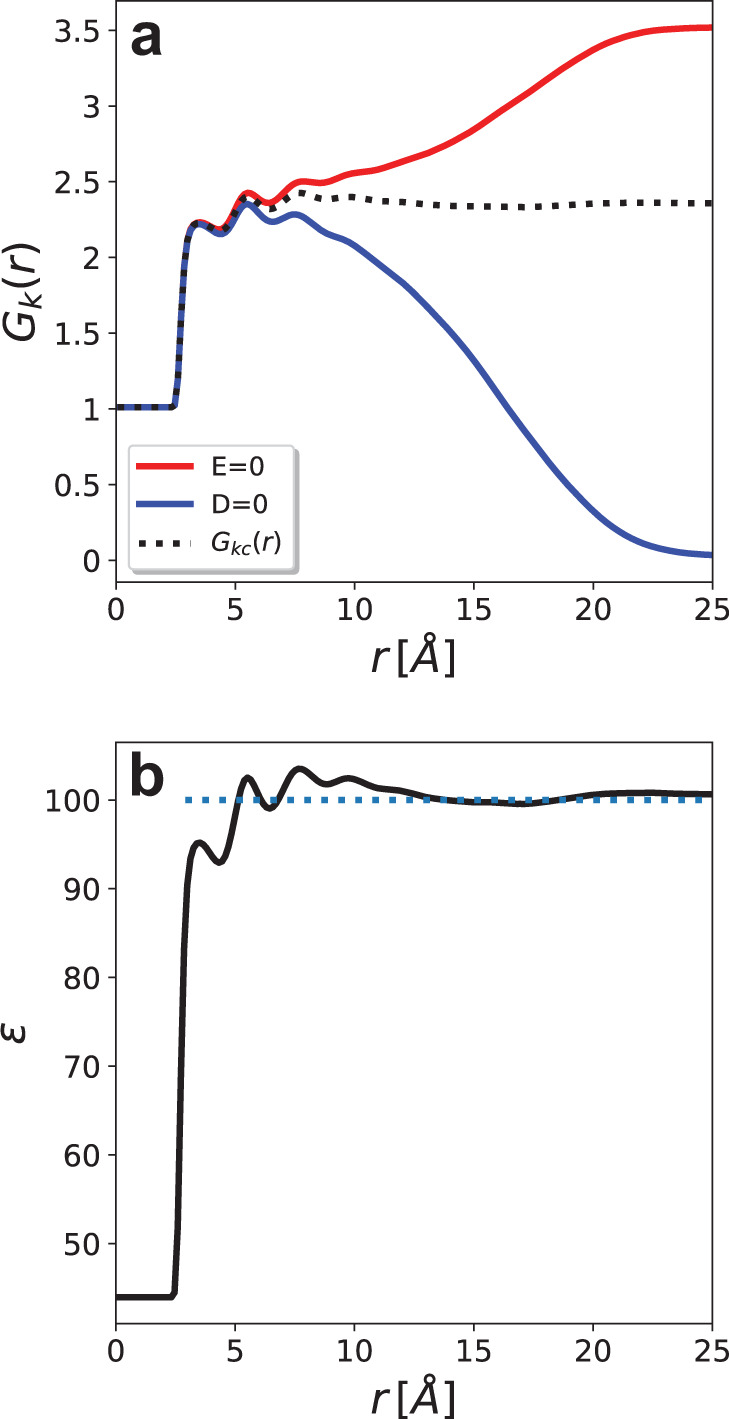
Estimating the dielectric constant. **a** The Kirkwood *g*-factors for zero electric field, **E** = 0, zero displacement field, **D** = 0, and the composite correlation function, *G*_Kc_(*r*), obtained from their superposition. **b** The effective distance-dependent dielectric constant obtained from *G*_Kc_(*r*) for the SCFNN model of water.

**μ**_1_ is the dipole of a water molecule at the origin and **M**_1_(*r*) is the total dipole moment in a sphere of radius *r* including the molecule at the origin. The composite Kirkwood *g*-factor,

5 $${G}_{{{{{{{{\rm{Kc}}}}}}}}}(r)=\frac{1}{3}\left[2{G}_{{{{{{{{\rm{K}}}}}}}}}{(r)}_{{{{{{{{\bf{E}}}}}}}} = 0}+{G}_{{{{{{{{\rm{K}}}}}}}}}{(r)}_{{{{{{{{\bf{D}}}}}}}} = 0}\right],$$

converges rapidly with *r* to a constant *g*_K_, which is related to the dielectric constant through Kirkwood’s relation for polarizable molecules^56^

6 $$\frac{4\pi \beta N{\mu }^{2}{g}_{{{\rm{K}}}}}{V}=\frac{(\varepsilon -1)(2\varepsilon +1)}{\varepsilon }-\frac{({\varepsilon }_{\infty }-1)(2{\varepsilon }_{\infty }+1)}{{\varepsilon }_{\infty }},$$

where *N* is the number of water molecules, *V* is the system volume, *β* = 1/(*k*_B_*T*), and *ε*_*∞*_ is the high-frequency dielectric constant that arises from electronic polarization; *ε*_*∞*_ ≈ 1.65 for the SCFNN model, as discussed below. As shown in Fig. 5a, the composite correlation function plateaus to a constant value near a distance of 6 Å, as expected^56^. By replacing *g*_K_ in Eq. (6) with *G*_Kc_(*r*) and inverting, we can compute the effective distance-dependent dielectric constant, shown in Fig. 5b. The dielectric constant rapidly converges to the bulk value of *ε* ≈ 100, which is close to estimates provided by van der Waals corrected functionals of similar accuracy^14^ and significantly less than that predicted by the PBE functional that overstructures water^56^.

**Fig. 6:**
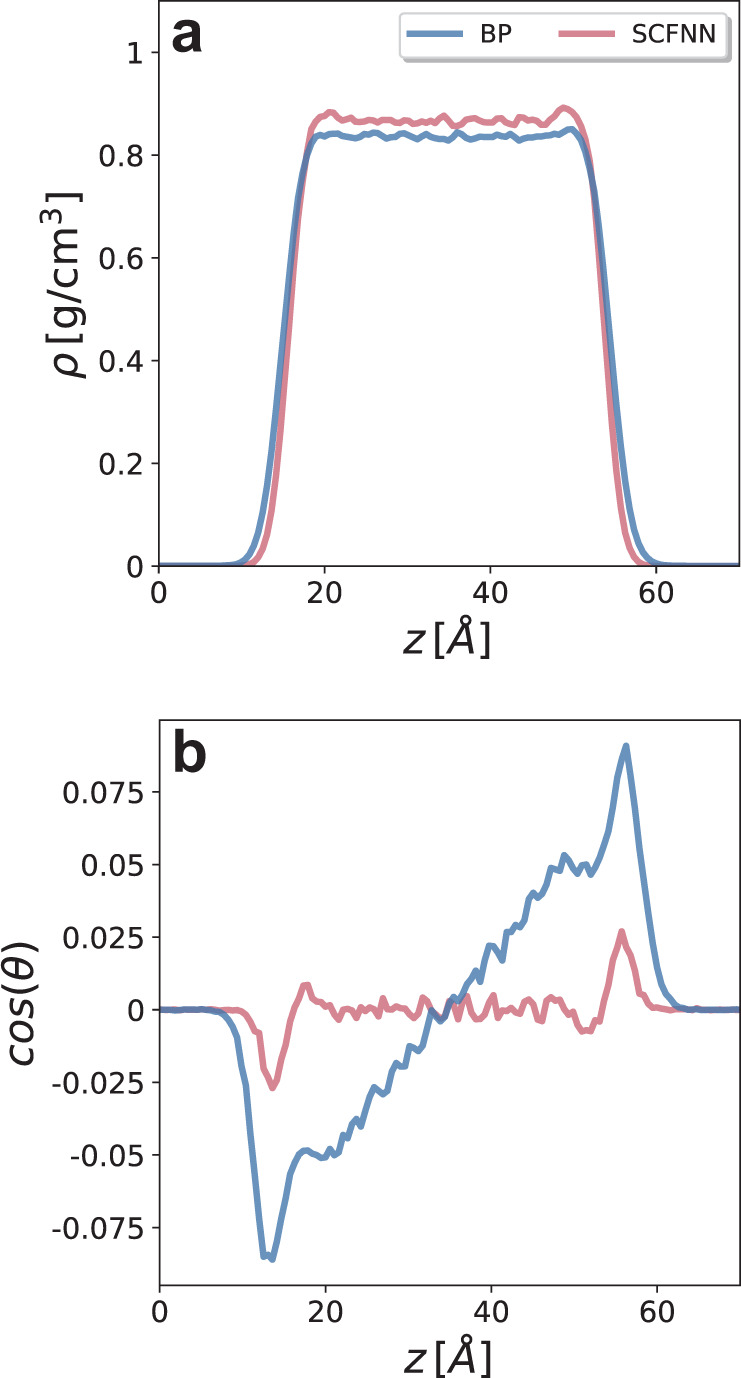
The structure of the water-vapor interface. **a** Water density and **b** average cosine of the angle formed by the water dipole moment and the surface normal for the Behler–Parrinello (BP) and SCFNN models without any additional training.

To push the limits of the SCFNN model, we can ask if it can properly predict dielectric screening in nonuniform environments, for which it was not trained. To do so, we simulate a water-vapor interface by extending the simulation cell along the *z*-axis to create a slab of water surrounded by a large vacuum region on either side. Because we have only trained on bulk configurations and not on configurations in the nonuniform system, we cannot expect the BP or the SCFNN model to accurately reproduce all features of the interface. Yet, both models do produce a stable interface, as shown by the densities in Fig. 6a, although the width of the SCFNN interfaces is smaller than those of the BP. Both models predict densities that are lower than those predicted by models explicitly trained for the interface, which may be expected because the bulk models did not learn the unbalanced dispersion forces that exist at interfaces^25,36,57^. However, the bulk density predicted by the SCFNN model is larger than that of the BP model, in better agreement with experiments.

Dielectric screening manifests in the orientational structure of interfacial water, and we examine the orientational preferences of water by computing $\langle {\cos} {\theta} ({z})\rangle$, where *θ*(*z*) is the angle formed by the surface normal and the dipole moment vector of a water molecule located at *z*. At the water–vapor interface, water molecules tend to point their dipoles slightly toward the vapor phase, a consequence of breaking an average of one H-bond per molecule at water’s surface. This dipole layer is screened by subsequent layers of water, such that no net orientation and zero electric field exists in the bulk. In the absence of long-range electrostatics, this screening is not achieved, and short-range models result in extended ordering from the interface into the bulk^25,36,37,39,44^. Indeed, the short ranged BP model results in long-ranged orientational ordering of water at the liquid–vapor interface because it lacks dielectric screening. In contrast, the SCFNN model displays the expected behavior. A single $\langle {\cos} {\theta} ({z}) \rangle$ peak in $\langle {\cos} {\theta} ({z}) \rangle$ appears near the interface and goes to zero in the bulk of the slab due to proper screening of the interfacial dipole layer. This successful prediction suggests that the SCFNN approach may lead to the creation of NN models that are at least partially transferable to different environments.

### Electronic fluctuations

**Fig. 7:**
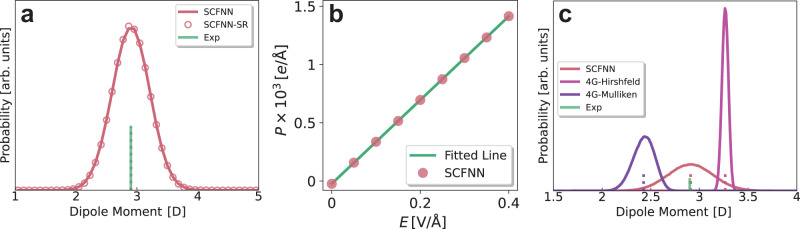
Electronic fluctuations in bulk water. **a** Probability distribution of the water dipole moment in bulk simulations of the SCFNN water model. Vertical solid and dashed lines indicate the average value estimated from experiments and the simulations, respectively. Data points indicate dipole moments predicted using the short-range network (SCFNN-SR). **b** Induced polarization as a function of the applied electric field with fixed nuclear configurations, which characterizes the high-frequency, electronic response of water to external fields. The solid line indicates predictions from dielectric theory used to estimate the high-frequency dielectric constant of the SCFNN model. **c** Probability distribution of the water dipole moment obtained using SCFNN and 4G-HDNNP models for the same set of nuclear configurations. Results for the 4G-HDNNP model are shown for both Hirshfeld and Mulliken charges. The set of configurations is from a bulk simulation of the SPC/E water model. Vertical dashed lines indicate the average dipole moment of each distribution.

In addition to the screening encompassed by the static dielectric constant, the SCFNN model can also properly predict electronic fluctuations of water and the high-frequency dielectric constant. To quantify electronic fluctuations, we compute the probability distribution of the magnitude of the water dipole moment from our simulations of bulk water using the SCFNN model, Fig. 7a. This distribution is dominated by the electronic polarization of water molecules and has a width consistent with predictions from ab initio MD simulations^58–61^. Moreover, the mean of the distribution yields an average dipole moment (2.9 D) in agreement with that estimated from experiments (2.9 D)^62^, further supporting that the SCFNN produces an accurate description of the molecular charge distribution in liquid water.

We also decomposed the dipole moment distribution into contributions from short- and long-range interactions. The short-range contribution to the electronic polarization is determined by Network 1S and the long-range part is determined by Network 1L. As shown in Fig. 7a, the molecular dipole moment distribution in bulk water is determined by short-range interactions, where the nuclear configurations of the bulk were determined using the full SCFNN model. This is consistent with the idea that local structure in a uniform bulk liquid, and fluctuations about that local structure, are determined by short-range interactions.

Long-range electronic effects on electrostatic screening are quantified by the high-frequency dielectric constant, *ε*_*∞*_. Physically, *ε*_*∞*_ can be thought of as the amount by which an electric field is screened without altering the positions of the nuclei; it quantifies the electronic response to applied fields. To estimate *ε*_*∞*_, we perform precisely this exercise: we compute the polarization of water in response to an external electric field of magnitude *E* and keep all positions of the nuclei fixed. The resulting polarization, shown in Fig. 7b, is consistent with a linear response to the field, as expected for dielectric screening. Fitting the induced electronic polarization to dielectric continuum theory expectations yields *ε*_*∞*_ ≈ 1.65, in good agreement with the experimental value of 1.77, demonstrating that the SCFNN model can accurately predict long-range electronic response to electrostatic fields.

Finally, we compare the electronic fluctuations of the SCFNN model to predictions made by the 4G-HDNNP model. To do so, we perform a MD simulation of bulk water using the extended simple point charge (SPC/E) water model^63^ and use the resulting configurations to determine the dipole moment distribution using each NN model, Fig. 7c. Using the same set of configurations allows us to compare only the ability of each model to predict charge distributions.

The 4G-HDNNP relies on atomic partial charges obtained from electronic structure calculations during the training process. The original implementation of the 4G-HDNNP model used Hirshfeld charges^28,64^. We additionally train another version of the 4G-HDNNP model using Mulliken charges to examine the dependence of the results on the method of determining the atomic partial charges^65^. See the Methods section for a more detailed discussion of the training procedure.

The SCFNN model results in a dipole moment distribution centered near the experimentally-determined average dipole moment. Moreover, the width of the SCFNN distribution is in good agreement with ab initio predictions^58–61^, although slightly narrower than that obtained using SCFNN-generated configurations (Fig. 7a). The 4G-HDNNP models result in significantly narrower distributions than the SCFNN model, and the average molecular dipole moment is either too large (Hirshfeld) or too small (Mulliken). The prediction of distinctly different molecular dipole moments demonstrates a key disadvantage of relying on atomic partial charges during training—the definition of partial charges can be ambiguous and often artificial. Then, the resulting 4G-HDNNP models trained with different partial charges will give different results. In contrast, the SCFNN model removes this ambiguity by representing the molecular charge distributions using MLWFCs.

## Discussion

In this work, we have presented a general strategy to construct NN potentials that can properly account for the long-range response of molecular systems that is responsible for dielectric screening and related phenomena. We demonstrated that this model produces the correct long-range polarization correlations in liquid water, as well as the correct response of liquid water to external electrostatic fields. Both of these quantities are related to the dielectric constant and require a proper description of long-range interactions. In contrast, current derivations of NN potentials result in short-range models that cannot capture these effects.

We anticipate that this approach will be of broad use to the molecular machine learning and simulation community for modeling the electrostatic and dielectric properties of molecular systems. In contrast to short-range interactions that must be properly learned to describe the different local environments encountered at extended interfaces and at solute surfaces, the response of the system to long-range, slowly-varying fields is quite general. Learning the long-range response (through Networks 1L and 2L) is analogous to learning a linear response in most cases, and we expect the resulting model to be relatively transferable; we emphasize, however, that the SCFNN is not limited to the linear response regime. As such, our resulting SCFNN model can make predictions about conditions on which it was not trained. For example, we trained the model for electric fields of magnitude 0, 0.1, and 0.2 V/Å, and then used this model to successfully predict the response of the system to displacement fields with magnitudes between 0 and 0.4 V/Å. This suggests that our approach can be used to train NN models in more complex environments and then accurately predict the response of water to long-range fields in those environments. We also showed that the SCFNN model trained for bulk water can predict orientational structure at the water-vapor interface as a result of learning dipolar screening, further emphasizing the ability of the SCFNN to predict the response of the system to electrostatic fields. The ability to learn the response of condensed phases to applied fields should make the SCFNN appealing for modeling atomic systems in electrochemical environments^66^, where electrostatic potential differences drive chemical processes, as well as in the modeling of interfaces with polar surfaces where the application of displacement fields is used to properly model surface charge densities^67,68^.

Our SCFNN approach is complementary to many established methods for creating NN potentials. Learning the short-range, GT system interactions can be accomplished with any method that uses local geometric information, and recent advances in optimizing this training can be leveraged^69,70^. In this case, the precise form of Networks 1S and 2S can be replaced with an alternative NN. Then, Networks 1L and 2L can be used as defined here, within the general SCFNN workflow, resulting in a variant of the desired NN potential that can describe the effects of long-range interactions. Because of this, we expect our SCFNN approach to be transferable and readily interfaced with current and future machine learning methods for modeling short-range molecular interactions.

We close with a discussion of the limitations of the SCFNN model in its current form and possible strategies for improvement. We rely on defining a local molecular coordinate system on each water molecule, in order to make our model rotationally equivariant. Moreover, we assumed that a specific number of MLWFCs are associated with each molecule, four for each water molecule, and examined their coordinates within the local frame. These steps are complicated when bond breakage and formation occurs. Although the general procedure can be readily extended to many molecules, the set of possible molecules must be known in advance. Strategies for constructing rotationally equivariant NN potentials without a local reference frame have been developed and can be used in place of the strategy used here to develop further generations of the SCFNN model that improve upon these deficiencies^30,48,71–73^.

## Methods

### Training the SCFNN

Our training and test set consists of 1571 configurations of 64 water molecules^52^. Homogeneous electric fields were applied to the system, as described further in the next section. We used two-thirds of the configurations for training and one-third to test the training of the network.

To train the networks we need to separate the DFT data into the GT system and the long-range effective field. However, that separation is not straightforward in practice. To achieve this, we use the differences in the MLWFC locations and forces induced by different fields to fit Networks 1L and 2L. We now describe this procedure in detail for fitting Network 1L, and Network 2L was fit following a similar approach.

To learn the effects of long-range interactions, we consider perturbations to the positions of the MLWFCs induced by external electric fields of different magnitudes. Consider applying two fields of strength $\left|{{{{{{{\bf{E}}}}}}}}\right|$ and $\left|{{{{{{{\bf{E}}}}}}}}\right|^{\prime}$. These fields will alter the MLWFC positions by Δ**r**_*w*_[**R**, **E**] and ${{\Delta }}{{{{{{{{\bf{r}}}}}}}}}_{w}^{\prime}[{{{{{{{\bf{R}}}}}}}},{{{{{{{\bf{E}}}}}}}}^{\prime} ]$, respectively. However, both Δ**r**_*w*_ and ${{\Delta }}{{{{{{{{\bf{r}}}}}}}}}_{w}^{\prime}$ are not directly obtainable from a single DFT calculation. Instead, we can readily compute the difference in perturbations, ${{\Delta }}{{{{{{{{\bf{r}}}}}}}}}_{w}-{{\Delta }}{{{{{{{{\bf{r}}}}}}}}}_{w}^{\prime}$, directly from the DFT data, because

7 $${{\Delta }}{{{{{{{{\bf{r}}}}}}}}}_{w}-{{\Delta }}{{{{{{{{\bf{r}}}}}}}}}_{w}^{\prime}={{{{{{{{\bf{r}}}}}}}}}_{w}-{{{{{{{{\bf{r}}}}}}}}}_{w}^{\prime}\,.$$

Here **r**_*w*_ and ${{{{{{{{\bf{r}}}}}}}}}_{w}^{\prime}$ are the locations of the MLWFCs in the full system in the presence of the field **E** and ${{{{{{{\bf{E}}}}}}}}^{\prime}$, respectively, and these positions can be readily computed in the simulations. These differences in the MLWFC positions are used to fit Network 1L. In addition, we also exploit the fact that Δ**r**_*w*_ = 0 when **E** = 0. This allows us to fix the zero point of Network 1L.

After fitting Networks 1L and 2L, we use them to predict the contribution of the effective field to the MLWFC locations and forces. We then subtract that part from the DFT data. What remains corresponds to the short-range GT system, and this is used to train Networks 1S and 2S.

We now describe the detailed structure of the four networks used here.

#### Network 1S

In the local frame of water molecule *i*, we construct two types of symmetry functions as inputs to Network 1S. The first type is the type 2 BP symmetry function^74^,

8 $${G}_{i}^{2}=\mathop{\sum}\limits_{j\ne i}\exp (-\eta {({r}_{ij}-{r}_{s})}^{2}){f}_{{{{{{\rm{c}}}}}}}({r}_{ij}).$$

Here *η* and *r*_*s*_ are parameters that adjust the width and center of the Gaussian, and *f*_c_ is a cutoff function whose value and slope go to zero at the radial cutoff *r*_c_. We adopted the same cutoff function as previous work^49^, and the cutoff *r*_c_ is set equal to 12 Bohr.

The second type of symmetry function is similar to the type 4 BP symmetry function^74^. This symmetry function depends on the angle between **r**_*ij*_ and the axis of the local frame,

9 $${{{{{{{{\bf{G}}}}}}}}}_{i}^{4}=\mathop{\sum}\limits_{j\ne i}{2}^{1-\zeta }{\left(1+\lambda \frac{{{{{{{{{\bf{r}}}}}}}}}_{ij}}{{r}_{ij}}\right)}^{\zeta }\exp (-\eta {r}_{ij}^{2}){f}_{{{{{{\rm{c}}}}}}}({r}_{ij}).$$

Here, *ζ* and *λ* are parameters that adjust the dependence of the angular term.

We use 36 symmetry functions as input to Network 1S. Network 1S itself consists of two hidden layers that contain 24 and 16 nodes. The output layer consists of 12 nodes, corresponding to the three-dimensional coordinates of the four MLWFCs of a central water molecule. Network 1S is a fully connected feed-forward network, and we use $\tanh (x)$ as its activation function.

#### Network 1L

In the local frame of water molecule *i*, we construct one type of symmetry function as input to Network 1L,

10 $${{{{{{{{\bf{EG}}}}}}}}}_{i}^{2}=\mathop{\sum}\limits_{j}{{{{{{{{\bf{E}}}}}}}}}_{j}\exp (-\eta {({r}_{ij}-{r}_{s})}^{2}){f}_{c}({r}_{ij}).$$

Here, **E**_*j*_ is the effective field exerted on atom *j*. We use 36 symmetry functions as inputs to Network 1L. Network 1L has no hidden layers. The output layer consists of 12 nodes, corresponding to the three-dimensional coordinates of the perturbations of a water molecule’s four MLWFCs induced by the external field.

#### Network 2S

Network 2S is exactly the same as the BP Network employed in the previous work^49^. In brief, the network contains 2 hidden layers, each containing 25 nodes. Type 2 and 4 BP symmetry functions are used as inputs to the network. The network for oxygen takes 30 symmetry functions as inputs, while the network for hydrogen takes 27 symmetry functions as inputs. A hyperbolic tangent is used as the activation function.

#### Network 2L

Network 2L uses the same type of symmetry function as Network 1L. The network for the force on the oxygen and for the force on hydrogen are trained independently. To predict the force on the oxygen, we center the local frame on the oxygen atom. When the force on a hydrogen atom is the target, we center the local frame on a hydrogen atom. We use 36 symmetry functions as inputs to Network 2L. Network 2L has no hidden layers. The inputs map linearly onto the forces on the atoms.

### 4G-HDNNP

The same configurations used to train and test the SCFNN model are used to train and test the 4G-HDNNP. Hirshfeld and Mulliken charges for these configurations are obtained with DFT. Two-thirds of these configurations are used to train the 4G-HDNNP and the remaining one-third is used to test the training. We trained two versions of the 4G-HDNNP, one with Hirshfeld charges and the other with Mulliken charges. The 4G-HDNNP-Hirshfeld model yields an average charge error of 0.012*e*_0_ on the test set, while the 4G-HDNNP-Mulliken yields an average charge error of 0.02*e*_0_ on the test set.

### DFT calculations

The DFT calculations followed previous work^52,75^ and used published configurations of water as the training set^52^. In short, all calculations were performed with CP2K (version 7)^76,77^, using the revPBE0 hybrid functional with 25% exact exchange^15,78,79^, the D3 dispersion correction of Grimme^80^, Goedecker–Tetter–Hutter pseudopotentials^81^, and TZV2P basis sets^82^, with a plane wave cutoff of 400 Ry. Maximally localized Wannier function centers^83^ were evaluated with CP2K, using the LOCALIZE option. The maximally localized Wannier function spreads were minimized according to previous work^84^. Hirshfeld and Mulliken charges were determined using the default implementations in CP2K. A homogeneous, external electric field was applied to the system using the Berry phase approach, with the PERIODIC_EFIELD option in CP2K^56,85,86^. Electric fields of magnitude 0, 0.1, and 0.2 V/Å were applied along the *z*-direction of the simulation cell. Sample input files are given at Zenodo^87^.

### MD simulations

MD simulations are performed in the canonical (NVT) ensemble, with a constant temperature of 300 K maintained using a Berendsen thermostat^88^. The system consisted of 1000 water molecules in a cubic box 31.2 Å in length. The equations of motion were integrated with a timestep of 0.5 fs. Radial distribution functions and longitudinal polarization correlation functions were computed from 100 independent trajectories that were each 50 ps in length. Finite-**D** simulations were performed under the same simulation conditions, and each trajectory was 50 ps long at each magnitude of *D*.

The liquid–vapor simulation was performed at 300 K. The system consisted of 1000 water molecules. The dimensions of the simulation box were *L*_*x*_ = *L*_*y*_ = 30 Å and *L*_*z*_ = 90 Å. The density profiles and the orientational profiles of water were obtained from 59 independent trajectories that were each 50 ps in length. Each trajectory is equilibrated for at least 50 ps before data are collected.

The SPC/E water^63^ simulation is performed in the canonical (NVT) ensemble, with a constant temperature of 300 K maintained using a Berendsen thermostat^88^. The system consisted of 1000 water molecules in a cubic box of length 31.2 Å. One thousand configurations were sampled from a 50 ns long trajectory of the SPC/E water simulation and the SCFNN and 4G-HDNNP were applied to these configurations to predict the dipole moments of water molecules.

## Data Availability

The data generated to train and test the SCFNN and the 4G-HDNNP have been deposited in Zenodo under accession code 10.5281/zenodo.5760191^87^. Source data are provided with this paper.

## Source data

Source Data

## Code availability

All DFT calculations were performed with CP2K version 7. In-house code was used to construct the NN potentials and perform the MD simulations. These codes are available on Github: 10.5281/zenodo.5919317^89^.
